# Efficacy and safety of laparoscopic splenectomy for hypersplenism secondary to portal hypertension after transjugular intrahepatic portosystemic shunt

**DOI:** 10.1186/s12876-021-01647-2

**Published:** 2021-02-11

**Authors:** Yingying Li, Zuojin Liu, Chang’an Liu

**Affiliations:** 1 Department of Hepatobiliary Surgery, Chongqing Medical University Affiliated Second Hospital, 74 Linjiang Road, Yuzhong District, Chongqing, China; 2 Second Clinical College, Department of Surgery, Chongqing Medical University, 1 Medical College Road, Yuzhong District, Chongqing, China

**Keywords:** Transjugular intrahepatic portosystemic shunt, Laparoscopic splenectomy, Portal hypertension, Hypersplenism

## Abstract

**Background:**

Laparoscopic splenectomy (LS) being used after Transjugular intrahepatic portosystemic shunt (TIPS) has not been reported. This report aims to explore the feasibility, safety, and potential efficacy of LS after TIPS hypersplenism secondary to portal hypertension (PHT).

**Methods:**

We retrospectively reviewed a series of six patients who underwent LS after TIPS for hypersplenism secondary to PHT between 2014 and 2020. The perioperative data and patients’ clinical outcomes were recorded.

**Results:**

LS was successfully performed in all patients. Hypersplenism was corrected after LS in all six patients. Postoperative prothrombin time, prothrombin activity, international normalized ratio, and total bilirubin showed a trend toward improvement. The preoperative and 1-month postoperative albumin and activated partial thromboplastin levels showed no significant difference. Plasma ammonia level and thromboelastography indicators were ameliorated in two limited recorded patients. No postoperative complications such as subphrenic abscess, portal vein thrombosis, variceal bleeding, hepatic encephalopathy, and liver failure occurred during the 1-month follow-up period.

**Conclusion:**

LS following TIPS is feasible, safe, and beneficial for patients with hypersplenism secondary to PHT. The following LS not only corrects the hypersplenism, but also has the potential to improve liver function.

## Background

In China, many patients have portal hypertension (PHT) secondary to liver cirrhosis as a result of the high incidence of chronic hepatitis B virus infection. PHT leads to many complications including gastroesophageal varices, ascites, splenomegaly, hypersplenism, coagulation abnormality, and hepatic dysfunction. An ideal treatment for PHT should aim at decreasing the portal venous pressure, controlling variceal bleeding, avoiding a recurrence of hemorrhage, correcting hypersplenism, maintaining a low rate of hepatic encephalopathy (HE), and improving liver function. However, there is no single therapy that could meet all the above requirements up to now.

Transjugular intrahepatic portosystemic shunt (TIPS) is mainly used in recurrent and refractory variceal hemorrhage and refractory ascites secondary to PHT [1]. However, there has no consensus been reached on the effect of TIPS in treating hypersplenism. Sanyal et al. reported TIPS had no effect on thrombocytopenia despite the portal pressure was decompressed [2]. Nevertheless, Massoud and Zein found TIPS may improve thrombocytopenia in liver cirrhotic patients [3]. These previous studies have reached conflicting conclusions. And no current clinical guidelines recommend TIPS as a treatment method for hypersplenism. Moreover, TIPS also brings a series of additional complications. The most life-threatening complications after TIPS include HE, heart failure, and liver failure [4].

Splenectomy is the preferred treatment for hypersplenism and has been recommended in China as an important treatment for PHT for a long time [5]. As a minimally invasive procedure, laparoscopic splenectomy (LS) is superior to open procedure in terms of less blood loss, lower operative complications, earlier resumption of oral intake, and shorter postoperative stay [6]. Splenectomy was also reported to improve the liver function in patients with splenomegaly [7]. Nevertheless, splenectomy alone does not solve the problem of recurrent and refractory variceal bleeding. It is reported the 5-year and 10-year recurrent bleeding rate after the procedure is 6.2% and 13.3% respectively [8]. Furthermore, splenectomy also brings the risk of portal vein thrombosis (PVT) [9, 10].

LS being used after TIPS in the same patient has not been reported in the English literature. Considering the above-mentioned advantages and disadvantages of LS and TIPS, we speculate the combined application of two procedures could produce a better effect on patients with hypersplenism secondary to PHT, comparing with the single use of any procedure. Therefore, six patients who received LS after TIPS were enrolled in this study. Our study aims to explore the feasibility, safety, and potential efficacy of LS following TIPS.

## Methods

After receiving institutional review board approval, a retrospective chart review was conducted on six patients who underwent LS after TIPS in our hospital between March 2014 and June 2020. All patients were diagnosed with PHT and hepatitis B virus-related cirrhosis. Preoperative abdomen computed tomography, color Doppler ultrasound, and gastroscopy were conducted to evaluate the severity of PHT and gastroesophageal varices. Color Doppler ultrasound was also performed to assess the TIPS shunt patency before LS. The indications for LS were as follows: patients with PHT and gastroesophageal varices; thrombocytopenia and/or leucopenia (white blood cell (WBC) count < 2.0 × 10^9^/L, platelet (PLT) count < 30 × 10^9^/L); at least one episode of esophagogastric variceal bleeding history; previous TIPS operation history; age greater than 18 years; general condition satisfying the surgery needs. The exclusion criteria included Child–Pugh C, combination with HE, and untreatable hepatocellular carcinoma.

After LS, all patients received low-molecular-weight heparin, followed by long-term clopidogrel or dipyridamole as prophylaxis therapy for PVT and shunt thrombosis. Preoperative data collection included age, sex, Child–Pugh score, Child–Pugh grade, the gap time between TIPS and LS, size of the spleen, and history of varices bleeding. All patients were followed up at seven days, and one month after LS. Follow-up laboratory examinations included blood routine tests, coagulation function tests, liver function tests, plasma ammonia level, and thromboelastography tests. Color Doppler ultrasound was performed to examine the PVT and the TIPS shunt patency. Postoperative complications were recorded during the 1-month follow-up period.

## Result

The basic clinical character of the six patients before LS is summarized in Table 1. Most patients are male (83%), mean age (47.5 ± 6) (mean ± standard deviation). The average Child–Pugh score was 6.8 ± 1.2 points, resulting in four (67%) Child–Pugh B and two (23%) Child–Pugh A patients. Mean WBC, PLT, and hemoglobin levels were 3.1 ± 1.2 × 10^9^/L, 43.7 ± 23.4 × 10^9^/L, and 82.3 ± 19.3 g/L respectively. The mean size of the spleen was 18.3 ± 2.9 cm. Gastroscopy confirmed serious esophagogastric varices in four (67%) patients and moderate esophagogastric varices in two (23%) patients (according to the grading systems of esophagogastric varices of China in 2008 [11], moderate esophagogastric varices is defined as straight or slightly winding varices with red signs or serpent-form varices without red signs; severe esophagogastric varices is defined as serpent-form varices with red signs or varices with a string of beads appearance, nodular appearance or tumor-like appearance with or without red signs). Three patients (67%) had one episode of variceal bleeding. Three (50%) patients experienced two episodes of variceal bleeding including one episode after receiving TIPS. All patients received only one TIPS procedure before LS, and the average gap time between TIPS and LS was 15.6 ± 10 months.

**Table 1:** The postoperative clinical character of the six patients

Case number	Year	Sex	Age	Child–Pugh score	Child–Pugh grade		WBC (× 10^9^/L)	PLT (× 10^9^/L)	HB (g/L)
Case1	2014	M	42	5	A		1.98	60	98
Case2	2014	M	40	8	B		3.86	67	66
Case3	2016	F	51	7	B		1.68	29	102
Case4	2016	M	58	6	A		3.65	24	125
Case5	2017	M	45	7	B		2.64	25	89
Case6	2019	M	49	8	B		2.82	23	74

Case number	Size of spleen (cm)		Gastroscopy findings			History of bleeding		Gap time^a^ (months)	
Case1	18.0 × 6.0		Serious esophagogastric varices			2		28	
Case2	17.5 × 7.3		Serious esophagogastric varices			2		10	
Case3	21.3 × 7.0		Moderate esophagogastric varices			1		8	
Case4	13.8 × 5.0		Moderate esophagogastric varices			2		26	
Case5	17.4 × 7.2		Serious esophagogastric varices			1		18	
Case6	21.7 × 7.2		Serious esophagogastric varices			1		4	

WBC: White blood cell; PLT: Platelet; HB: Hemoglobin

^a^Gap time refers to the gap time between transjugular intrahepatic portosystemic shunt and the following laparoscopic splenectomy

**Fig. 1:**
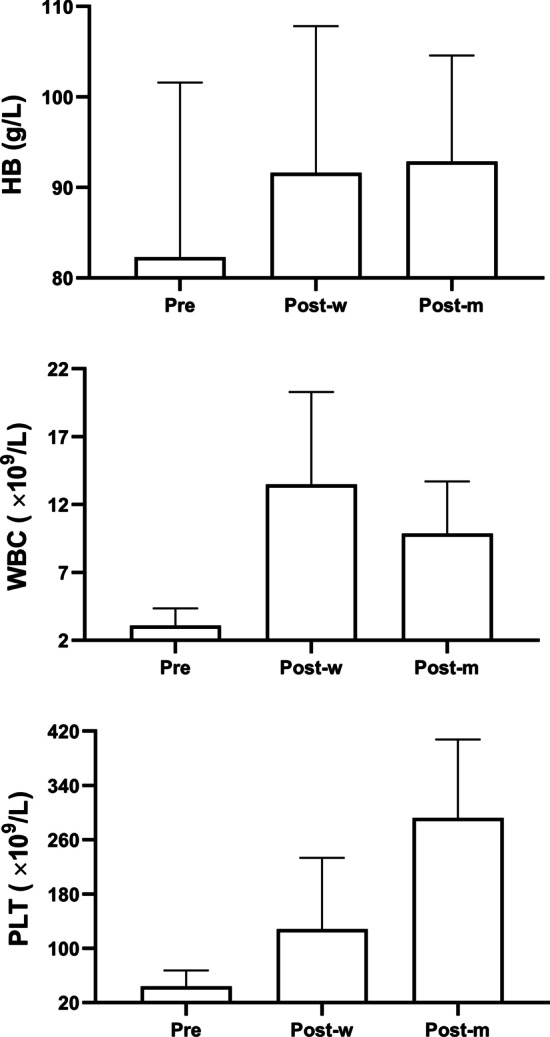
Changes of blood routine test results before and after surgery. Hemoglobin (HB), white blood cell (WBC), and platelet (PLT) were tested respectively. The average levels of each group were as shown in the bar graphs, and groups were as labeled under X axis (*Pre*, preoperative; *Post-w*, 7-days postoperative; *Post-m*, 1-month postoperative)

**Fig. 2:**
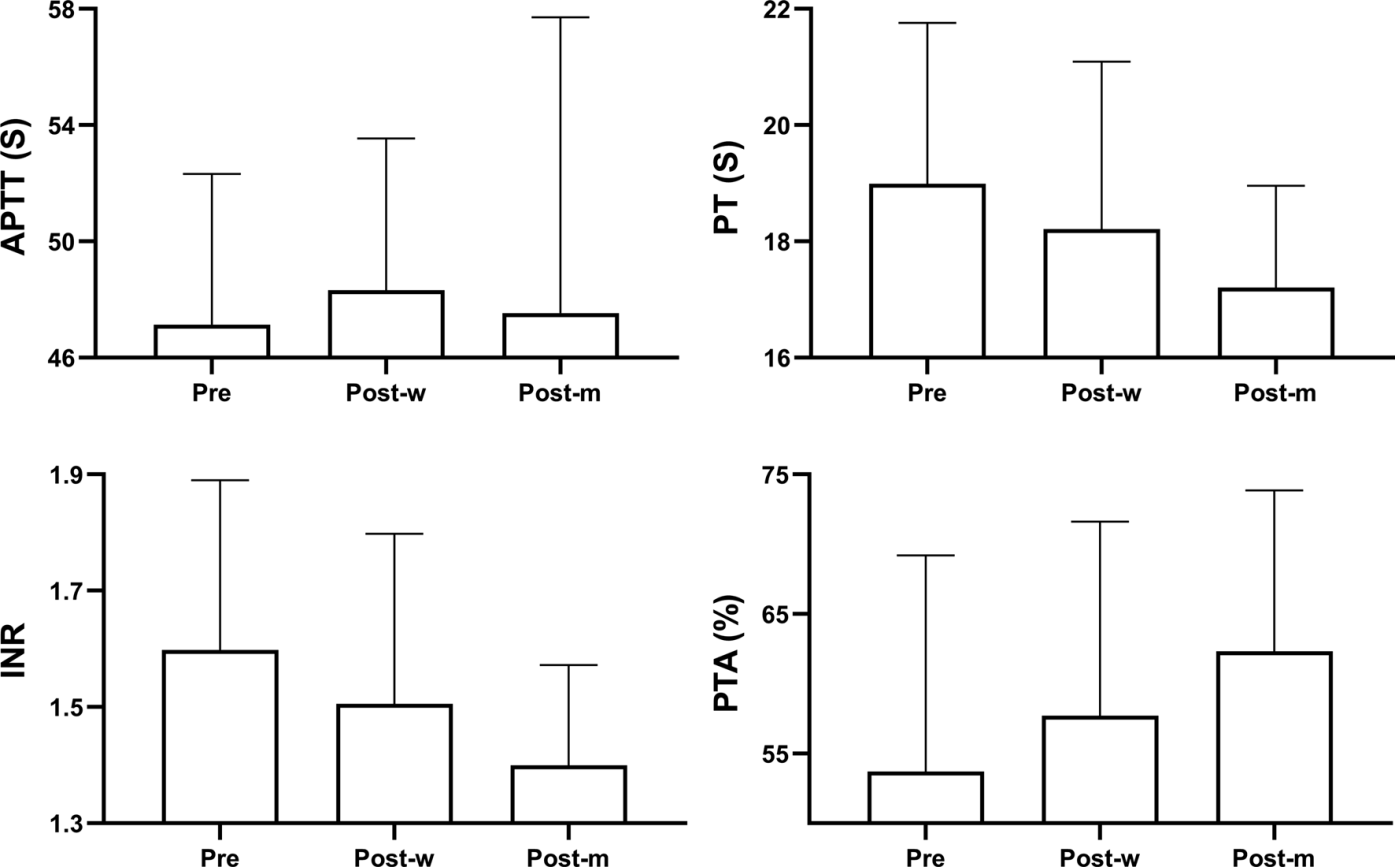
Changes of coagulation test results before and after surgery. Prothrombin time (PT), activated partial thromboplastin (APTT), international normalized ratio (INR), prothrombin activity (PTA) were tested respectively. The average levels of each group were as shown in the bar graphs, and groups were as labeled under X axis (*Pre*, preoperative; *Post-w*, 7-days postoperative; *Post-m*, 1-month postoperative)

**Fig. 3:**
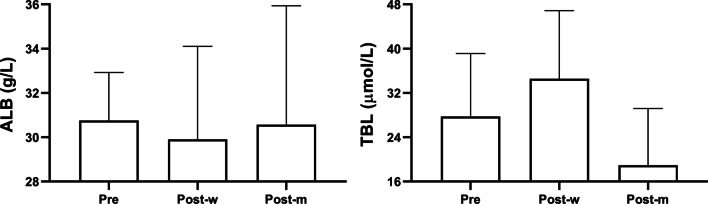
Changes of liver function test results before and after surgery. Albumin (ALB), and total bilirubin (TBL) were tested respectively. The average levels of each group were as shown in the bar graphs, and groups were as labeled under X axis (*Pre*, preoperative; *Post-w*, 7-days postoperative; *Post-m*, 1-month postoperative)

The clinical laboratory results from 7-days preoperatively to 1-month postoperatively are presented in Figs. 1, 2 and 3. Hemoglobin, WBC, and PLT were elevated 1-month after LS (Fig. 1). The mean prothrombin time, international normalized ratio, prothrombin activity levels also showed an improvement trend, though non-statistically significant. No significant change was observed in activated partial thromboplastin (Fig. 2). The mean serum total bilirubin level was raised 1-month after LS, yet the albumin level remained unchanged (Fig. 3). Only two limited patients had the record of plasma ammonia level and thromboelastography test results. The postoperative plasma ammonia level was reduced in both two patients. The maximum amplitude value, R value, K time, and α angle also showed improvement in both patients.

The Child–Pugh score was increased in three (50%) patients and remain unchanged in one (17%) patient. The mean Child–Pugh score was reduced from 6.8 ± 1.2 to 6.3 ± 0.8 points, resulting in three (50%) Child–Pugh B and three (50%) Child–Pugh A patients. All six patients recovered well and were discharged within 15 days postoperatively. No serious complications such as subphrenic abscess, portal vein thrombosis, stent stenosis or occlusion, variceal bleeding, HE, and liver failure occurred in any patients during the 1-months follow-up period.

## Discussion

In our study, hypersplenism was corrected after LS in patients who underwent TIPS before. In addition to increasing the PLT count, LS can also improve the quality of PLT by elevating the maximum amplitude value. The liver function was also ameliorated after LS. The plasma ammonia level was decreased in two limited recorded patients. The improvement of hypersplenism and liver function after the single use of LS corresponds well with the previous studies. A study from China suggested TIPS procedure is safe and effective for variceal bleeding with chronic portal vein occlusion after splenectomy, which revealed the feasibility of the combining use of TIPS and splenectomy [12]. However, the efficacy of LS following TIPS has not been reported in the English literature. To our knowledge, our study is the first report to explore the value of LS after TIPS.

Early TIPS was proven to be superior to drugs plus endoscopic procedure in the treatment of acute variceal hemorrhage secondary to PHT, through reducing failure to control bleeding and further bleeding rate, decreasing new or worsening ascites, and improving transplantation-free survival [13–15]. TIPS is also highly feasible, effective, and safe for PVT recanalization according to a recent systematic review with meta-analysis [16]. Furthermore, TIPS stent insertions were found to effectively prevent rebleeding in cirrhotic patients with PVT and variceal hemorrhage history. Considering the high incidence of PVT after splenectomy, it is interesting to explore whether TIPS is also effective in preventing PVT in patients who underwent LS before. Although, more evidence is needed to support the assumption.

Despite plenty of advantages of TIPS, TIPS brings a series of additional complications. The most life-threatening one is HE. HE mainly due to a fraction of the unfiltered portal flow directly into the hepatic vein (portosystemic shunts) [17]. Portosystemic shunt from TIPS stent insertion mainly participates in post-TIPS HE. This kind of shunt can be achieved by shunt reduction or occlusion techniques, such as using a small size TIPS stent [18] or placing a parallel balloon-expandable stent inside the traditional TIPS stent [19]. Meanwhile, spontaneous portosystemic shunt (SPSS) has gained recent recognition for its important role in HE. In several studies, large SPSS was found to increase the risk for HE and mortality in patients with cirrhosis [20–22]. Laleman et al. substantiated the embolization of large SPSSs had efficacy for recurrent HE [23]. Among all kinds of SPSSs, splenorenal shunts were the most common, representing 32–41% of cirrhotic patients with SPSS [20]. LS is a radical surgery for splenorenal shunts. Thus, we speculate whether LS could also decrease the rates of post-TIPS HE by diminishing portosystemic shunt. In our study, postoperative plasma ammonia level was decreased in two patients, which may indicate LS has a positive impact on post-TIPS HE. However, this remains to be further confirmed by a larger study.

The impact of TIPS on liver function has not reached a consensus. Jalan et al. reported the liver function tests and indocyanine green clearance showed temporary deterioration after TIPS [24]. An American study suggested the hepatobiliary laboratory values (bilirubin, aspartate aminotransferase, alanine aminotransferase, alkaline phosphatase, and international normalized ratio) showed significant increases in a short term after TIPS compared with baseline levels [25], which means the deterioration in liver function. However, studies also demonstrated unchanged or positive results of liver function after TIPS [26, 27]. For patients with poor liver function after TIPS, the possible causes may be the direct mechanical impairment to the hepatic parenchyma during TIPS creation as well as potentially decreased intrahepatic portal venous flow and resultant hepatocytes ischemia [25].

On the contrary, splenectomy has been proven to ameliorate liver function in patients with liver cirrhosis. In several studies, the prothrombin activity, prothrombin time, and serum levels of total bilirubin significantly improved one year after splenectomy, resulting in decreased Child–Pugh scores [28, 29]. Studies also suggest that splenectomy can enhance liver synthesis function and reduce liver fibrosis [30, 31]. Our findings are consistent with prior studies, in which the coagulation function and liver function were ameliorated after LS, and the Child–Pugh scores decreased in 50% of patients. Our study implied that succeeding LS may also improve the post-TIPS liver function in cirrhotic patients, which needs to be verified by further research.

The underlying mechanism of the improved liver after splenectomy has not been fully elucidated. In a rat model, splenectomy caused a rise in hepatic arterial blood flow with sufficient oxygen supply to the liver [32]. Thus, the increased intrahepatic blood perfusion to the hepatic artery may better nourish hepatocytes. Meanwhile, the postoperatively changed cytokines after splenectomy, including decreased TGF-β1 and elevated IL-6 produced in splenic tissue, may also contribute to the favorable effect on liver fibrosis. Akahoshi et al. found that spleen-derived TGF-β1 inhibiting the regeneration of the damaged liver in an animal experiment [33]. Asanoma et al. found the expression of IL-6 in the spleens of cirrhotic patients was significantly lower than noncirrhotic patients and concluded that lower expression of IL-6 resulting from splenomegaly may inhibit liver regeneration [34]. However, more evidence is needed to corroborate the positive role of splenectomy in liver fibrosis.

The effect of TIPS in treating hypersplenism is controversial. Here we mainly focus on the most common clinical manifestation of hypersplenism, thrombocytopenia. Jabbour et al. reported TIPS was ineffective for thrombocytopenia even though the portosystemic gradient was decreased to less than 12 mmHg [35]. The study by Barney et al. suggested PLT was increased during the first three months after TIPS but returned to the level before TIPS by 12–14 months [36]. Karasu et al. even found PLT had a non-statistically significant tendency to reduce [37]. However, studies also found TIPS may improve the thrombocytopenia associated with liver cirrhosis, but no correlation was proved between the changes in portal pressure gradient and PLT count [38, 39]. The potential mechanism of TIPS improving hypersplenism may be reducing the portosystemic gradient and improving the PHT, resulting in alleviating splenic congestion. Nevertheless, a study by Gschwantler et al. has suggested PHT is just a minor influencing factor in thrombocytopenia [40]. Other factors such as low serum levels of thrombopoietin, translocated toxins or other gut-derived substances, anti-platelet antibodies also play important roles in thrombocytopenia [41–43]. Thus, no current clinical guidelines recommend TIPS as a treatment method for hypersplenism.

Splenectomy is a major treatment for hypersplenism. General indications for splenectomy are malignancy, and hematological autoimmune disorders [44]. Whether splenectomy is applicable to treat the hypersplenism secondary to PHT remains disputed. Some scholars conceive that splenectomy brings high risks of surgical bleeding and serious postoperative complications and should be avoided in patients with PHT [45]. However, splenectomy is commonly performed and recommended in China for patients with PHT. As an invasive technique, LS has recently received increasing attention and favor for its multiple benefits. LS has been proven to safe and effective in treating PHT [46]. Current guideline stands that LS only applies to normal-sized or moderately enlarged spleens and massive splenomegaly is regarded as a potential contraindication for LS [47]. This opinion is mainly based on the fact that the operating space is limited for laparoscopic operation in a massive spleen situation and the rate of conversion to open splenectomy is rather high. On the contrary, some studies found LS is feasible and safe for even massive or supermassive spleens with improved laparoscopic expertise and advancing technology [48–50]. Casaccia et al. found LS has significantly less blood loss, shorter hospital stays, and experienced no conversion in patients with massive and giant spleens. They thought that the size of the spleen as the only parameter is insufficient to determine the availability of laparoscopic approach. The actual abdominal “working space” based on the abdominal dimensions and body mass index should also be taken into consideration [51]. In our study, the intraoperative hemorrhage was less than 200 ml in all patients while the average diameter of the spleen was 18.3 ± 2.9 cm. All patients recovered well and were discharged within two weeks. No serious complications occurred during our follow-up period. Our results demonstrated again that LS is safe and feasible in patients with PHT.

Partial splenic embolization (PSE) is another optional therapy for hypersplenism. Comparing with LS, PSE is a simple, rapid, mini-invasive procedure and exhibits less intraoperative bleeding, shorter operative time, and shorter length of hospital stay in the treatment of hypersplenism in liver cirrhotic patients [52]. Furthermore, PSE retains the repeatability of operation and preserves adequate splenic tissue to prevent overwhelming infection [53]. Alzen et al. even considered PSE should be performed as an alternative to splenectomy since PSE is free from the risk of overwhelming post-splenectomy infection syndrome (OPSI) [54]. However, the improvement of hypersplenism only lasted for 6 months after PSE when the splenic infarction rate was less than 50% and may need repeated phases of PSE [55], which would result in increasing the health care costs and surgical burden on patients. Moreover, PSE would cause abscess formation and liver failure when the infarction rate is more than 70%, which may lead to death in 1% of patients [56]. Although there is a risk of OPSI after splenectomy, the rate of OPSI mainly depends on age, indication for splenectomy, and ongoing immunosuppression. Splenectomy performed for PHT and in adult patients presents a relatively low incidence of OPSI [57]. In addition, LS can reduce portal venous pressure and eradicate the spontaneous splenorenal shunts. As a result, splenectomy remains the first choice of hypersplenism secondary to PHT in China.

It is noticeable that the combined use of TIPS and PSE is also safe and feasible. Wan YM et al. found that the followed PSE after TIPS may improve the long-term primary shunt patency rate and markedly reduce the shunt dysfunction rates. But the overall survival was not significantly different between TIPS group and combined use of TIPS and PSE (TIPS + PSE) group [58]. Li YH et al. also found that TIPS + PSE is superior to TIPS alone in controlling variceal rebleeding and shunt stenosis [59]. The portal venous pressure was proven to be significantly decreased after PSE in both studies. TIPS + PSE was also found to be feasible in Child C class patients. However, the efficacy of TIPS + PSE on hypersplenism was not explored in any report and requires further studies to confirm.

This study has several limitations as follows: First, the small sample size and retrospective nature are clear limitations of our study. Second, the long-term efficacy of LS following TIPS remains unclear since the follow-up period was only one month. Third, the selection of patients for LS is restrictive, for LS does not apply to Child–Pugh C patients.

## Conclusion

In conclusion, LS after TIPS is feasible and safe in patients with hypersplenism secondary to PHT. Regarding a curative effect of LS after TIPS, our study showed that LS not only corrects hypersplenism, it can also improve the liver function in a short term. Furthermore, LS may have a positive impact on post-TIPS HE by diminishing spontaneous splenorenal shunt. The long-term efficacy of LS after TIPS will require further investigation in larger and longer prospective studies.

## Data Availability

The datasets generated during and/or analyzed during the current study are not publicly available due to the confidentiality of human subjects but are available from the corresponding author on reasonable request.

## Abbreviations

LS: Laparoscopic splenectomy
TIPS: Transjugular intrahepatic portosystemic shunt
PHT: Portal hypertension
HE: Hepatic encephalopathy
PVT: Portal vein thrombosis
WBC: White blood cell
PLT: Platelet
SPSS: Spontaneous portosystemic shunt
PSE: Partial splenic embolization
OPSI: Overwhelming post-splenectomy infection syndrome
